# Decision-making on maternal pertussis vaccination among women in a vaccine-hesitant religious group: Stages and needs

**DOI:** 10.1371/journal.pone.0242261

**Published:** 2020-11-12

**Authors:** Anne C. de Munter, Wilhelmina L. M. Ruijs, Robert A. C. Ruiter, Dagmar J. J. van Nimwegen, Anke J. M. Oerlemans, Rijk van Ginkel, Marlies E. J. L. Hulscher, Jeannine L. A. Hautvast

**Affiliations:** 1 Department of Primary and Community Care, Radboud Institute for Health Sciences, Radboud University Medical Center, Nijmegen, The Netherlands; 2 Department of Infectious Disease Control, GGD Gelderland-Zuid, Nijmegen, The Netherlands; 3 Department of Work & Social Psychology, Maastricht University, Maastricht, The Netherlands; 4 IQ healthcare, Radboud Institute for Health Sciences, Radboud University Medical Center, Nijmegen, The Netherlands; 5 “Fruits of Passion; Together Fighting Human Suffering”, Bodegraven, The Netherlands; Brigham Young University, UNITED STATES

## Abstract

**Introduction:**

As of December 2019, pregnant women in the Netherlands are offered pertussis vaccination to protect their newborn infant against pertussis infection. However, the manner in which pregnant women decide about this maternal pertussis vaccination is largely unknown. The aim of this study is to gain insight into the decision-making process regarding maternal pertussis vaccination, and to explore the related needs among the vaccine-hesitant subgroup of orthodox Protestant women.

**Methods:**

Charmaz’s grounded theory approach was used to develop a decision-making framework. To construct this framework we used an explorative multimethod approach in which in-depth interviews and online focus groups were supplemented by a literature search and research group meetings. This study was carried out in a hypothetical situation since the maternal pertussis vaccination had yet to be implemented in the Dutch immunisation programme at the time of the study.

**Results:**

Twenty-five orthodox Protestant women participated in an interview, an online focus group, or in both. The findings of this study resulted in a decision-making framework that included three stages of decision-making; an Orientation stage, a value-based Deliberation stage, and Final decision stage. The Orientation stage included the needs for decision-making categorised into Information needs and Conversation needs. Women indicated that -if they were to receive sufficient time for Orientation and Deliberation- they would be able to reach the stage of Final decision.

**Conclusion:**

The decision-making framework resulting from our findings can be used by health care professionals to provide women with information and consultation in the decision-making process. Future studies should investigate whether the stages of and needs for decision-making can be found across other vaccine-hesitant subgroups and vaccinations.

## Introduction

Pertussis is a highly contagious respiratory disease, characterized by severe coughing spells [1, 2]. Especially in young infants, pertussis can cause complications such as pneumonia, apnoea, and respiratory failure [3]. Pertussis vaccination is offered in most childhood vaccination programmes. However, the effectiveness of the acellular pertussis vaccine wanes over time, resulting in an increase of pertussis incidence in countries using this pertussis vaccine [4, 5]. The increasing pertussis incidence is most threatening for newborn infants, who are too young to be fully vaccinated.

Since maternal pertussis vaccination is a highly effective, safe and cost-effective intervention to prevent pertussis in newborn babies, many countries recommend pertussis vaccination for pregnant women [6–8]. However, regardless of the general public and individual health benefits of the maternal pertussis vaccine, in various countries which provide maternal pertussis vaccination for pregnant women, health care professionals (HCPs) and governments are confronted with parents’ vaccine hesitancy and lower vaccine uptake among pregnant women than expected [9–12]. Providing information and education on the maternal pertussis vaccination does not seem to be sufficient in addressing these women’s doubts, as hesitance remains after being informed by an HCP [13, 14].

As of December 2019, the maternal pertussis vaccination is included in the Dutch immunisation programme. However, in the Netherlands, similar to other Western countries, various groups of people question or refuse vaccinations due to ideological, philosophical or religious beliefs [15–19]. One of these vaccine-hesitant groups is the Dutch orthodox Protestant community which comprises 1.5% of the Dutch population. This community has a long history of vaccine hesitancy as, since the introduction of vaccinations in the 19^th^ century, orthodox Protestants have raised religious objections to vaccinations [20]. Research findings indicate that orthodox Protestant parents make a well-considered decision about childhood vaccinations and do not consider accepting vaccinations as self-evident [18]. Childhood vaccination coverage in this community ranges between 11% and 86%, depending of which specific church denomination orthodox Protestants are member of [21, 22].

The hesitance towards vaccination in general and maternal pertussis vaccination in particular underlines the importance of shaping the decision-making process in such a way that the information and decision support needs of vaccine-hesitant parents are taken into account [23–25]. Hitherto, however, little is known about vaccine-hesitant parents’ needs regarding decision-making on maternal pertussis vaccination [11, 26–28].

The aim of this study is to gain insight into the decision-making process on the newly introduced maternal pertussis vaccination, and the related needs among a vaccine-hesitant group, namely Dutch orthodox Protestant women. This knowledge can then be used by HCPs to optimize their assistance and support and may contribute to less hesitancy regarding the maternal pertussis vaccination among this specific group of women.

## Methods

### Research design

**Fig 1:**
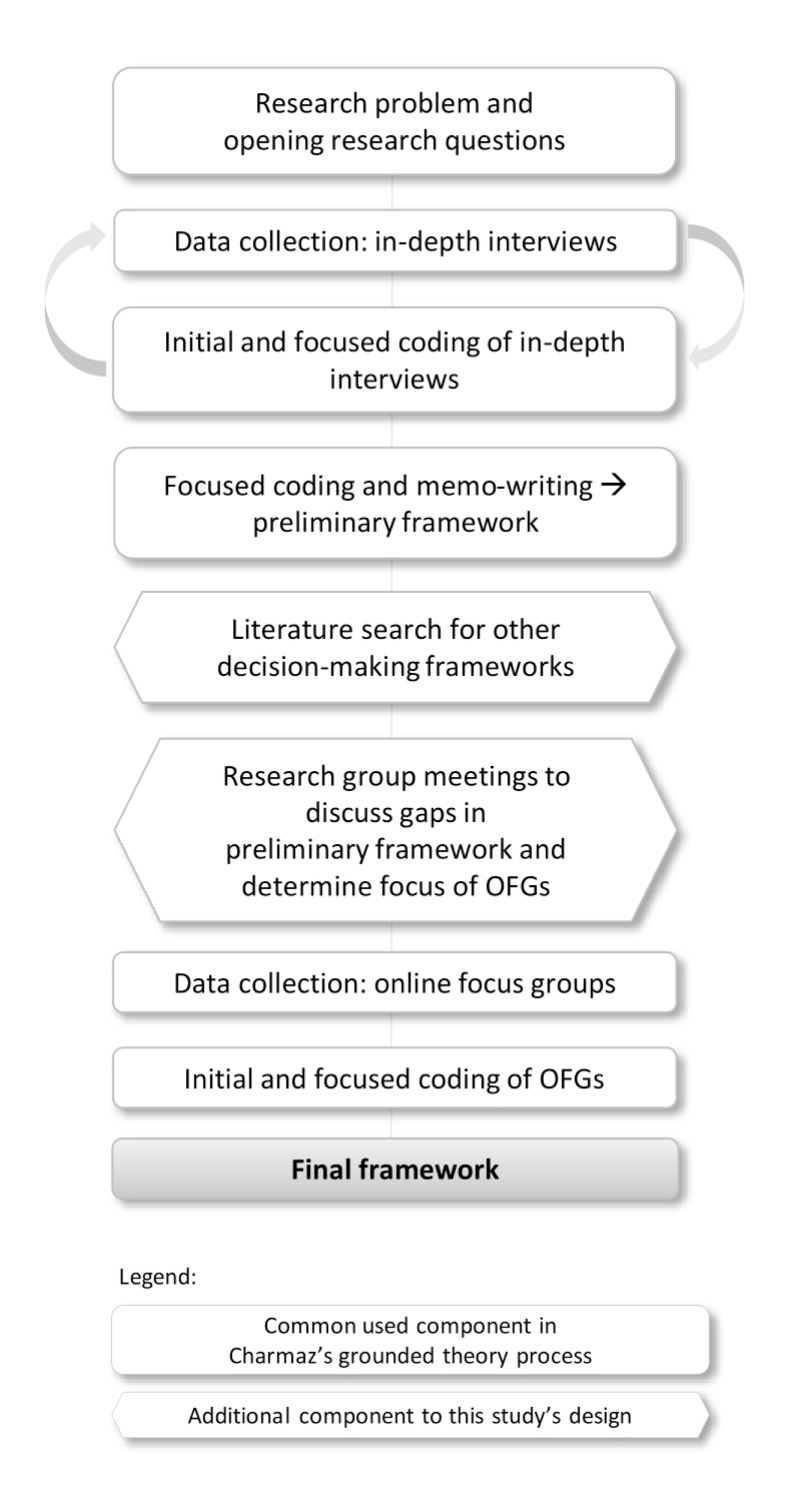
Flowchart of this study’s constructivist grounded theory method.

To explore the decision-making process and related needs of the participating women, a qualitative research design following Charmaz’s grounded theory approach was used to develop an analytic framework of the orthodox Protestant women’s decision-making process regarding maternal pertussis vaccination (Fig 1) [29]. Methodological triangulation, including interviews, online focus groups (OFGs), a literature search and research group meetings, was applied to increase the validity of the data [30]. Our data collection started with in-depth individual interviews using an inductive approach. Data analysis of the interviews revealed several stages and corresponding needs in decision-making, resulting in a preliminary decision-making framework. Next, a literature search was conducted to find comparable decision-making frameworks from earlier publications, to establish possible gaps in the preliminary framework and to spark new insights. Subsequently, research group meetings (co-authors AdM, WR, RR, MH and JH) were used to look at our preliminary framework from a bird’s-eye view and to discuss these gaps in our framework, taking into account how the OFGs could be used to close these gaps. Finally, OFGs were carried out to determine whether women recognised their decision-making process in the presented stages and corresponding needs as described in the framework, and to gain insight into potentially inconclusive topics. Results from the OFGs were used to refine the framework, resulting in the final framework.

### Study population and recruitment

The study population consisted of orthodox Protestant women aged between 18–40 years old. A purposeful sampling method was used. We explicitly selected participants aiming at a broad variety of women regarding pregnancy status, having children, age, membership to various orthodox Protestant church denominations, and residence in different regions of the Netherlands. Aim of this purposive sampling, as opposed to probability sampling, is to include ‘information rich cases for in-depth study’ [31]. As we aimed to explore the decision-making process of women who were expected to make a decision about maternal pertussis vaccination in the near future, we recruited married women, knowing that becoming pregnant generally is the wish of married couples of this religion. Key persons (individuals with close contacts within the orthodox Protestant community) provided entrance to the orthodox Protestant minority as this community is considered to be hard to reach. Key persons approached orthodox Protestant women to verify if they were willing to participate in the study, if so, the researchers would provide additional study information. Additionally, interviewed women were asked to approach other women from their network for participation in the study. This snowball sampling was used to reach women who could not be reached through key persons and to minimize selection bias by only including women of the key persons’ networks. All interviewees were asked to participate in an OFG, as it was considered to be of added value if OFG participants had previously reflected on their decision-making process in an interview. Besides, other eligible participants were approached to participate in an OFG using purposeful sampling via key persons and through snowball sampling via interviewees and participants who already joined an OFG.

After an initial telephone contact with one of the researchers (AdM or DvN), interviewees and OFG participants were provided with written information about the study objective, interview or OFG procedure, and the voluntary nature of participation. After one week the researcher contacted the potential participant to answer any questions the participant might still have had. After oral consent, an interview appointment was made or participation in an OFG was scheduled. Written consent was obtained before the start of the interview or OFG. Participants received a gift voucher for participation.

### Interviews

Semi-structured in-depth interviews were conducted between March and August 2017 by trained female interviewers (AdM, DvN and WR). The interviewers had knowledge of the orthodox Protestant community, yet were not members of an orthodox Protestant denomination. The interviews lasted 20–60 minutes, with a mean of 30 minutes. To create a confidential environment, interviews were held at the participants’ homes. The interview started with an introduction to the study objectives. A topic guide, which contained several general open-ended questions about the needs for decision-making and the decision-making process, was used to preserve a basic structure in the interviews (see Table 1 for the English translation of the topic guide). The topic guide was developed by the authors, and pretested in two pilot interviews; no adjustments were needed. If the participant was not familiar with the maternal pertussis vaccination, a short introduction was given on the subject (Table 1). Data were collected until data saturation was reached, after no new insights in the categories of the decision-making process or needs for decision-making emerged in the final interviews. Results from the interviews were used to shape the preliminary framework.

**Table 1:** Interview topic guide.

**Introduction: Familiarity with maternal pertussis vaccination**
• Have you ever heard of the pertussis vaccination for pregnant women?
If this is not the case, the interviewer provides the following introduction of the maternal pertussis vaccination: The Health Council advises to provide a pertussis vaccination to every pregnant woman in the Netherlands. This vaccination will be given between weeks 28 and 32* of pregnancy. In response to the vaccination, the pregnant woman’s immune system makes antibodies. These antibodies will be transferred to the fetus via the umbilical cord. Thus, the baby will be born with sufficient antibodies to protect him/her against pertussis during the first months of life.
**Decision-making process and needs**
• How would you make the decision regarding getting this vaccination?
• What do you need to make this decision?
• Would you discuss this vaccination with others? Who would this be and what would you like to discuss?
**Final decision**
• What is the most important reason for you to accept or decline the vaccination?
• Who makes the final decision?

* At the time of the interview and OFG data collection, the National Coordination Centre for Communicable Diseases Control of the National Institute for Public Health and the Environment advised pregnant women to receive the maternal pertussis vaccination between weeks 28 and 32 of pregnancy. In the current Dutch immunisation programme, pregnant women can receive the vaccination as from the twenty-second week of pregnancy.

### Literature search

Search terms used in PubMed were the MeSH terms “Vaccination” OR “Immunization” AND “Decision making”, combined with “Framework” OR “Stage” OR “Model” OR “Phase” OR “Process” OR “Steps”. The preliminary framework resulting from the interviews, and the frameworks following the literature search were discussed in research group meetings (with AdM, WR, RR, MH and JH), which resulted in a multistage decision-making framework, including needs for decision-making.

### Online focus groups (OFGs)

In the final study phase, focus groups were carried out in an online environment, which allowed us to include women from different geographical areas in the Netherlands, as it offered the possibility of overcoming geographical distance among participants. Women could remain anonymous by participating under a nickname, which was considered an advantage as vaccination may be a sensitive topic for orthodox Protestants and anonymity could reduce social desirability bias [18, 32]. We used asynchronous OFGs, meaning that participants did not have to be online at the same time. Therefore, women had the ability to reflect on our questions, the responses of other women, and the response they wanted to share [32].

In April and May 2018, three OFGs were conducted with a maximum of six participants per group. Before the start of each OFG, participants received written information about the maternal pertussis vaccination, instructions about the online forum, and a nickname and password. An OFG lasted from Monday to Friday, starting each morning with a ‘question of the day’.(See Table 2 for the English translation of the online focus group topic guide). Women were asked to answer the researcher’s questions and react to other participants’ responses. During the day, the researcher (AdM) would post a second or third question in response to the group discussion. Results from the OFGs were used to refine the framework until consensus was reached.

**Table 2:** Online focus group topic guide.

**Monday**	
Main Topic:	Information
Introduction:	You received information about the pertussis vaccination for pregnant women.
Questions:	What was your first reaction? What did you like about the information? What did you dislike about the information? Could you make a decision based on this information?
**Tuesday**	
Main Topic:	Health care professional and other people
Introduction:	Imagine you are pregnant and your midwife offers you the vaccination.
Questions:	How can your midwife support you in making a decision? Which support would you like to receive from another health care professional?
	Would you discuss the vaccination with someone other than the health care professional? And if so, who would this be?
**Wednesday**	
Main Topic:	Deliberation: Religious beliefs
Questions:	Do your religious beliefs play a role in your decision-making about the maternal pertussis vaccination? And if so, how are your religious beliefs involved in your decision-making?
**Thursday**	
Main Topic:	Other deliberation themes
Introduction:	Looking at your posts from past days, I noticed that you gather information, opinions and guidance in various ways, e.g. by searching the internet, discussing with family or friends, praying, and reading in the Bible.
Questions:	How do you weigh all this?
	Is it important for you to make a good decision? And do you know where this derives from? (only in OFG 2 and 3)
**Friday**	
Main Topic	Evaluation group meeting
Questions	How did you experience this online group discussion? Would an online forum or group meeting contribute to your decision-making? If there was a group meeting about the maternal pertussis vaccination in which information about the vaccination was shared and you could discuss the vaccination issue–would you go to such a meeting? Would it make any difference to you if this meeting was organised by a Christian organisation?

### Data analysis

Interviews were recorded with a digital voice recorder and transcribed verbatim. To provide anonymity, references to individuals were removed from the transcripts. Transcripts were analysed using the qualitative software program ATLAS.ti 8. Two researchers (AdM, DvN) conducted the initial coding of the interview transcripts independently, in which lines and segments of the transcripts were coded line-by-line and solely based on the content of the data. Initial codes were reviewed, discussed and refined (AdM, DvN, WR) until consensus was reached. Using focused coding, codes were combined into categories (AdM, WR) including stages and needs in the decision-making process. In the memo-writing process, these categories and codes were transformed into a preliminary framework.

After completion of the OFGs, all posted comments were adopted unchanged into the transcript. For the data analysis of the OFGs, two researchers (AdM, WR) analysed the OFG transcripts using initial and focused coding, taking into account that these codes and categories could differ from the interview codes and categories. Newly emerging codes and categories were discussed and refined until consensus was reached. Subsequently, emerging categories were compared with those in the preliminary framework, and used to adapt the framework.

This study adheres to the COREQ guidelines for reporting qualitative studies [33]. Quotes were translated from Dutch to English by the first and last author and then checked by an external bilingual reviewer.

### Ethics and privacy

The Medical Ethics Committee (CMO) of the Arnhem-Nijmegen region assessed the study in February 2017 and concluded that it was exempt from their approval; reference no. 2017–3178.

## Results

In total, 25 women participated in the study. Fifteen women participated in an individual interview and sixteen women took part in one of the three OFGs, of which six women had previously participated in an individual interview. Of the fifteen women participating in an individual interview, nine declined OFG participation, primarily because the OFG would be too time-consuming.

All participating women were married and most had children. Their children were either vaccinated, unvaccinated or partially vaccinated. Participant characteristics are shown in Table 3. Since the maternal pertussis vaccination was not yet implemented in the national immunisation programme at the time of the interviews and OFGs, participants were asked to hypothesize their future decision-making as if they were pregnant and had to decide on pertussis vaccination uptake.

**Table 3:** Participant characteristics of interviewees, interviewees who participated in an online focus group, and online focus group participants (total N = 25).

	Interviewees (n = 9)	Interviewees and OFG participants (n = 6)	OFG participants (n = 10)
**Years of Age (range)**	23–36	24–37	26–36
**Pregnancy status**			
Pregnant	4	n/a	1
Not pregnant	5	1	9
Pregnant during interview or OFG	n/a	5	n/a
**Children**			
Yes	8	5	9
No	1	1	1
**Children of participant are vaccinated**			
Yes	4	2	5
No or not intended to	4	3	2
Partially*	0	1	2
Unknown or not applicable	1	0	1
**Church denomination**			
High level of conservatism	4	1	0
Moderate level of conservatism	4	3	9
Low level of conservatism	1	2	1

* Partially vaccinated = some of the participant’s children were vaccinated and other children were not, and/or the participant’s children had not received all recommended vaccinations.

OFG = online focus group; n/a = not applicable

### Decision-making framework: Stages and needs

**Fig 2:**
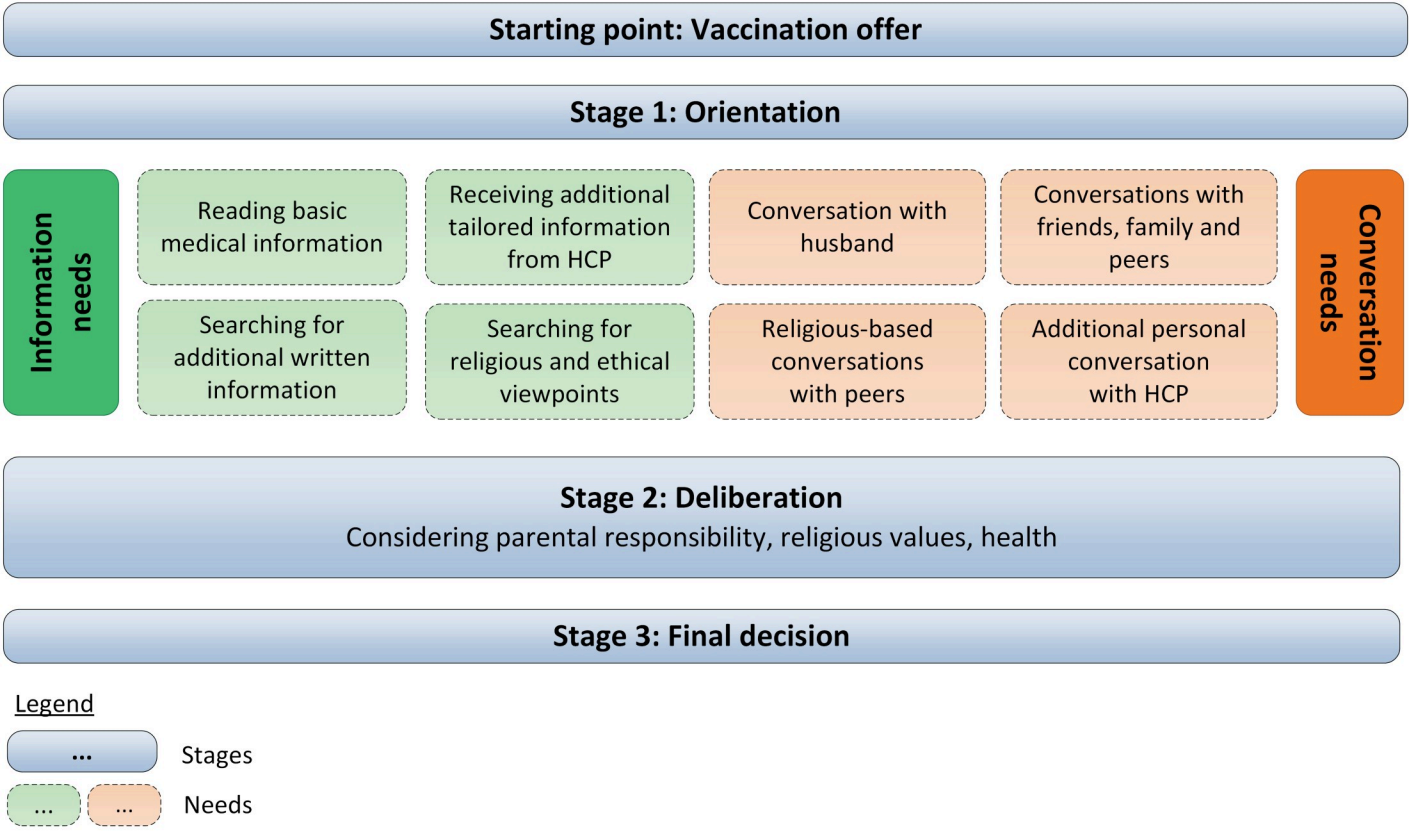
Framework of decision-making process on maternal pertussis vaccination among orthodox Protestant women.

A preliminary framework, which contained multiple stages and corresponding needs in the decision-making process, was constructed from the results of the individual interviews. After 15 interviews data saturation of the interviews was reached. The literature search resulted in three publications which visualized a vaccine decision-making framework: Bartolini et al. (2012), Brunson (2013), and McNeil et al. (2019) [34–36]. The models of Bartolini et al. (2012) and Brunson (2013) included separate stages of decision-making, yet, only the framework of Bartolini et al. (2012) included needs for decision-making in these stages. The model of McNeil et al. (2019) described decision-making as a deliberative process. These frameworks were used in the research group meetings to refine our preliminary framework. Lastly, after the analysis of the OFGs, we stated that we gathered sufficient information in the three OFGs to complete the framework (Fig 2).

In the paragraphs below, the interview and OFG results are described collectively, following the structure of the stages and needs in the final framework, illustrated by quotes of participants (Table 4). The starting point of the framework is the Vaccination offer from the HCP, followed by three stages of decision-making. Stage 1 in the decision-making process is Orientation, in which women gather information and discuss the topic with others. In stage 2, Deliberation, women contemplate the values they consider to be most important in this vaccination issue. Stage 3 encompasses the Final decision to accept or decline vaccination. Women mentioned specific needs in the decision-making process most often in relation to their Orientation. These needs are categorised into Information needs and Conversation needs.

**Table 4:** Quotes from orthodox Protestant women during interviews and online focus groups.

**Stage 1. Orientation**
**Information need: Reading basic medical information**
*“A bit of information about the new [vaccine]*. *You can receive a vaccination during your pregnancy and then what*? *Why would you do that*? *So*, *certain motivations*. […] *I would very much like to know what consequences it has for the baby in my womb*. *Whether it has negative consequences as well*, *and if this is the case*, *I just want to know the truth*.” (Interview 8)
*“If you have to make a decision based on this information* (information provided prior to the OFG), *then you almost feel it is mandatory*, *because you don’t have another choice*. *It is either vaccinating* (accepting the maternal pertussis vaccination) *or life-threatening* (if your newborn gets whooping cough).” (OFG1, woman 6)
**Information need: Receiving additional tailored information from HCP**
*“If there are reasons to make the advice personal*, *I would like this information to be provided by the midwife*, *as well*. *I am thinking about certain (risk)factors which I might have and should*, *therefore*, *be discussed*.*”* (OFG1, woman 4)
*She* (midwife) *is very professional and knows a lot*. […] *I consider her to be an expert*, *when it concerns babies*.(Interview 13)
**Information need: Searching for additional written information**
*“Because I am used to view everything from different angles and to read more about it before I come to a consideration*, *I would also search for additional information*. *I would request and read the package insert*. *Based on this I would continue my search; there is various information from different perspectives to be found on the Internet*.*”* (OFG1, woman 5)
*“The downside*, *I really find this a downside*, *because if you search on the Internet*, *which you actually shouldn’t do*, *because you find a lot of*, *a lot of information which you cannot always assess to be reliable*.*”* (Interview 8).
*“Now it is so explicitly pro*, *that if you also want to hear counterarguments*, *you must first research for this yourself*.*”* (OFG2, woman 9)
**Information need: Searching for religious and ethical viewpoints**
*“You read the* (orthodox Protestant) *newspaper*, *you read Terdege* (orthodox Protestant magazine), *you read*, *well*, *for example*, *this brochure from the NPV* (Dutch Christian patient organization) *that you receive*.*”* (Interview 13)
*“I would appreciate hearing about the ethical side*. *Especially in case of such a ‘new’ vaccination while the baby is still in the womb*, *I would really like to know more*!*”* (OFG2, woman 16)
**Conversation need: Conversation with husband**
*“We are on the same page about most things for that matter*, *fortunately*. *Yes*, *I think*, *I mean*, *you talk about it together*. *I think you search for information*, *or you read what you received somewhere*. […] *I would not look into this alone*, *no*. *It is really something you decide together*.*”* (Interview 3)
**Conversation need: Conversations with friends, family and peers**
*“I think I would mainly talk about it with others around me who are pregnant at that moment and who have to make the decision*, *or those who have just made the decision*.*”* (OFG2, woman 8)
*“We*, *Christians*, *can think we know everything*, *but of course that doesn’t have to be true*. *Sometimes you can learn*, *I think*, *from people who are not part of anything* (people who are not religious) *and who decide on other grounds*.*”* (Interview 5)
**Conversation need: Religious-based conversations with peers**
*“It is*, *of course*, *also the case that people from the orthodox Protestant minorities most often rely on their own groups*. […] *Then you talk about it with your own people*, *maybe also because*, *yes*, *that is where you come from; it feels familiar and the choices they make*, *also feel good for you*.*”* (Interview 7)
*“I would be curious to know if others with the same religious beliefs would*, *for example*, *have ethical arguments on why to do it or why not*. *Sheerly for my own consideration*.*”* (OFG2, woman 14)
**Conversation need: Additional personal conversation with HCP**
*“What I always like very much is*, *that it* (information given by the HCP) *comes with empathy and a sympathetic ear and good arguments*. […] *That means a lot to me*.*”* (Interview 14)
*“You don’t expect information on principle issues from the midwife*. […] *I don’t expect that such information can be compatible to our way of living and to our feelings and to our religious beliefs*.*”* (Interview 11)
**Stage 2. Deliberation**
**Parental responsibility**
*“In the end*, *I*, *myself*, *am responsible for the unborn child in the sense that I take care of my own body during pregnancy and in that way create circumstances that are as optimal as possible*. *I cannot impose that responsibility on someone else*.*”* (OFG1, woman 5)
*“Actually*, *the feeling/maternal intuition is nearly the most important in making a decision*.*”* (OFG3, woman 15)
**Religious values**
*“It is not the case that the Bible*, *which I read*, *says ‘Thou shalt not vaccinate’*. […] *I really believe that God has given me intellect*, *which I shall have to use to make wise choices*.*”* (Interview 12)
*“That is the ‘struggle’ for us*. *We have our own responsibilities and we may use the available resources*, *but that doesn’t mean that we blindly want to protect ourselves against everything (and*, *so*, *vaccinate)*. (OFG2, woman 9)
*“In my opinion*, *you should make the decision in dependence on God*, *knowing that God is above all*. *However*, *you have your responsibility and you should use the available means (if ethically justified)*.”(OFG2, woman 12)
**Health**
*“Then I would calculate the risks*. […] *Is the odds five thousand to one*, *or is the chance a hundred to one*? *Is the risk of a side-effect greater than its benefit*? *Or is the danger of not-vaccinating*, *for example*, *the chance that your child gets it* (pertussis), *or that your child dies because of it* (pertussis).” (Interview 12)
*“I try*, *with the resources available*, *healthy food and vitamins*, *to increase resistance*. *I’d do that rather than a vaccination*. *That is most important for me*.*”* (Interview 7)
**Step 3. Final decision**
*“It remains our own responsibility if you do it or not* (vaccinate) *and not because it is customary*.*”* (OFG1, woman 1)
*“In the end*, *it is not very relevant whether you do it or do not do it* (vaccinate), *but most of all that in your conscience you are certain that you did everything to receive the right information*.*”* (OFG3, woman 15)

OFG = online focus group; HCP = health care professional

### Starting point of the decision-making process: Vaccination offer

Most women were unfamiliar with the maternal pertussis vaccination. One pregnant interviewee did not need an introduction of the vaccination, as she already had read some information about it. She could not recall the source (HCP or other) of the information. Women presumed that their midwife would notify them about the vaccination in one of their regular appointments during pregnancy. Some women, who refused childhood vaccinations on religious grounds, pointed out that they would probably also refuse maternal vaccination. Nevertheless, they indicated that they would like to be informed about the vaccination offer and other possibilities to protect their unborn or newborn child against pertussis.

### Stage 1 in the decision-making process: Orientation

Participants presumed that they would have many questions following the Vaccination offer. Therefore, women considered they would need time to gather and read information and discuss their options with others, to satisfy their Information and Conversation needs. Some women, especially those who expressed more hesitancy towards the vaccination, expected that they would orient themselves more broadly and extensively than others.

#### Information needs in stage 1 Orientation

Participants’ initial information need was to receive basic information provided by HCPs. After which, women expected to manage the rest of their needs themselves; searching for information that they believed was relevant for them and discussing the vaccination issue with specific people in their surroundings.

*Reading basic medical information*. Women indicated they would first need basic, factual medical information that would answer their most pressing questions about pertussis and the vaccination, e.g. questions about vaccine safety and the necessity and effectiveness of the vaccine. Participants expected that this information would be provided in a brochure or website by the HCP or national public health service. They hoped that these brochures or websites would contain trustworthy, independent and non-directive information. However, mentioned by interviewees and supported by OFG participants, women expected the standard information would provide a one-sided viewpoint -one in favour of vaccination- and would contain insufficient information on which to base their decision.

*Receiving additional tailored information from HCPs*. Several participants wanted HCPs to tailor the information and/or motivation to vaccinate to their personal situation, e.g. if the participant had a medically complicated pregnancy or lived in a high- or low-risk area for pertussis incidence. Midwives were seen as the most trustworthy persons to provide this information. They were expected to have ample expertise about the topic and they would have the best interest for the unborn child and the pregnant woman in mind. Some participants thought that, in addition to their midwife, general practitioners (GPs), obstetrics clinic personnel and professionals from public health centres could also provide credible and solid information.

*Searching for additional written information*. Most participants indicated they would search for additional information on the Internet or in books. The Internet was assumed to create a complete information overview from different sources, however, some women stated they often found it difficult to make a selection of valid online information. Therefore, they would prefer a list of reliable, informative websites from HCPs. Websites of the national public health authority (RIVM) or midwife associations were thought to provide credible and essential medical information. Despite this, some women presumed that these websites might provide mainly positive aspects of the vaccination and, therefore, they would actively search for possible negative effects, and alternatives to vaccinations on websites from other sources, such as anti-vaccine movements and homeopathy practices.

*Searching for religious and ethical viewpoints*. To fulfil their needs for information on religious aspects of the vaccination, e.g., themes of divine providence, trust in God, and the responsibility of man, participants would seek for answers in the Bible and opinions written by representatives from their own religious constituency in books, on websites or in newspapers. Some women mentioned they highly valued the brochure from a Dutch Christian patient organisation, containing these main religious themes regarding vaccination. In addition, some women wanted to read more about the medical ethical view on the vaccination to determine its proportionate use in the prevention of illness, especially since the vaccination could influence the natural process of pregnancy given by God.

#### Conversation needs in stage 1 Orientation

Most participants felt the need to discuss the vaccination with others; their husband, friends, family, peers, neighbours, and acquaintances. In these conversations, they assembled opinions, information, and experiences about the vaccination issue.

*Conversation with husband*. Participants considered their husband to be the most important person to discuss the vaccination issue with since husband and wife should decide about the vaccination together. A few women would only discuss this vaccination issue with their husband, as they considered this decision to be a personal matter. Some couples would read medical and religious information together and/or pray together for God’s support in their decision-making.

*Conversations with family*, *friends and peers*. Conversation partners preferred by the participants, besides the husband, were women who stand close to them, who have to make the same decision or have an equal opinion about vaccinations. Several women pointed out that having a conversation with someone who has a contrasting opinion about vaccinations, or one who has a different religious background or no religious background at all, could create a more complete view about the matter. Face-to-face conversations were more often experienced as meaningful conversations, compared to online conversations. Most women thought that sharing their doubts, opinions and/or considerations in a group meeting would have an added value in their decision-making.

*Religious-based conversations with peers*. Discussions about religious and ethical topics were confined to one-on-one or group conversations with other orthodox Protestants or Protestant Christians. The aim of these conversations was to gather religious and/or ethical viewpoints or to serve as confirmation of one’s own opinion. Some women thought that group meetings about the maternal pertussis vaccination provided by a Christian organisation would give a more objective and nuanced (ethical) view on the vaccination, and these meetings were expected to be more considerate of their religious beliefs than a group meeting provided by the midwife practice.

*Additional personal conversation with HCP*. Several participants felt the need for an open, personal consultation with their HCP, which was not (primarily) based on receiving factual medical information. These women reckoned they would like to hear the HCP’s personal experiences with pertussis and/or personal viewpoints on the vaccination. However, both women who did and did not prefer a personal consultation with their HCP, disliked it when HCPs imposed their opinion on them following their strong desire to make a personal decision. In addition, HCPs were not expected to discuss religious views on the vaccination, since religious considerations are seen as a personal matter.

### Stage 2 in the decision-making process: Deliberation

Participants mentioned an overall need for a timeout moment during the decision-making process to contemplate the information and viewpoints they gathered in their Orientation stage, and the personal values they considered to be most important in their decision concerning this vaccination issue. In this Deliberation stage three themes of combined values could be identified: parental responsibility, religious values and health. Often various personal values coexisted in this Deliberation. However, some participants indicated that one main value could outweigh other values. For instance, many highly conservative orthodox Protestant participants mentioned they considered their trust in God (religious value) to be most important and, therefore, they might choose not to vaccinate. This could outweigh their understanding that the vaccination can prevent a pertussis infection (health). The findings did not indicate separate needs for decision-making in the Deliberation stage.

#### Parental responsibility

Most participants experienced a strong sense that, as parents, they are responsible for their child’s health, a responsibility that could not be entrusted to others. Particularly pregnant women and first-time mothers mentioned they considered it to be their primary role as an (expectant) mother to ensure a healthy pregnancy and protect their (unborn) child against all negative influences, if possible. A few experienced mothers presumed that they would choose to accept or decline vaccination based on their intuition, which would give them the feeling of ‘doing the right thing’ as a parent. Some women indicated they wanted to be able to explain their well-considered decision to their child in the future.

#### Religious values

The religious values regarding this vaccination topic could be divided in two main issues. On one hand, the issue of interfering with divine providence and absolute trust in God, including his plan regarding sickness and health. For some participants, accepting vaccination implied distrust in God’s protection and the life that is predetermined for them. In addition, some women considered vaccination an undesirable intervention in light of ‘human enhancement’ i.e. that mankind tries to outdo God.

On the other hand, women referred to the issue that God has given mankind the responsibility to use available knowledge to make a well-considered decision. Some women considered the vaccination a gift from God, as mankind was given the capability to develop vaccines to keep children healthy. Several participants expressed that they experienced a strong conflict between these religious values.

Ultimately, it was considered to be most important that a decision was made in line with one’s own religious beliefs, and one’s personal relationship with God.

#### Health

Participants would use the gathered information to weigh all health-related pros, cons, and alternatives to the vaccination which they considered relevant for their personal situation. Participants frequently mentioned that they aimed to strike a balance between their perceived risk and severity of pertussis, and their perceived benefit of the vaccine. The possible risks of the vaccines were mostly discussed in relation to the unknown negative influence it may have on the pregnancy and the unborn child. However, some participants stated that it was better if she, a healthy adult, would receive the vaccine, instead of their vulnerable newborn child. In contrast to women who tended to accept vaccination, hesitant women highly valued the alternatives to the vaccination. More hesitant participants emphasised the added value of preventing pertussis infection by breastfeeding your baby, healthy nutrition, and taking homeopathic products.

### Stage 3 in the decision-making process: Final decision

Even though this study was based on a hypothetical situation and women had not yet made a final decision, several participants mentioned their intention to either accept or decline this vaccination during pregnancy. Most women wanted to make a well-considered decision and indicated that, if they were given enough time for Orientation and Deliberation, they would be able to do so. A few women who objected to vaccinations on religious grounds expected their decision-making process to be concise, resulting in refusing the maternal pertussis vaccination. Overall, all women considered it to be of great importance that husband and wife would make the final decision together.

## Discussion

In this study, we distinguished stages and needs in the decision-making process regarding the newly introduced maternal pertussis vaccination in a vaccine-hesitant religious population. The framework, resulting from the findings in this study, describes the decision-making process and included an Orientation stage, a value-based Deliberation stage, and Final decision stage. The needs for decision-making are concentrated in the Orientation stage and are categorised into Information needs and Conversation needs.

In contrast to our study, conducted in a religious group in an European country, the studies on the decision-making process found in the literature search were carried out among general populations in North and South America [34–36]. The religious background of the participants and/or the influence of religion on the decision-making process within these studies was unknown. Nevertheless, in the frameworks and descriptions of the decision-making process provided in these publications, we found striking similarities in the processes of decision-making compared to the decision-making process of our study population [34–36]. Resembling results were also found in other publications, which did not contain a decision-making framework or model, yet, which did describe comparable elements in the decision-making process, as discussed below [26, 37–55]. This implicates that, even though our study is conducted among a vaccine-hesitant religious group in the Netherlands, our framework could be applicable to a broader population.

The desire of parents to actively orient themselves on the vaccination issue by fulfilling both information and conversation needs was broadly supported by other studies on vaccination decision-making [25, 34, 35, 37, 44, 52]. In our study, vaccine hesitant religious women were inclined to follow a thorough Orientation stage by actively searching for information and having conversations with others. Like Brunson’s ‘searchers’, they conduct their own research by seeking information from multiple pro- and contra-vaccination published sources and tend to be critical about the information they obtain [35]. Or like Wiley’s ‘proactive types’ they actively search for information and use their HCP as an information source [52]. Consistent with our results, HCPs are considered to be trustworthy information sources [37, 47]. Yet, compared to vaccine-acceptant parents, vaccine-hesitant parents are more critical about which information source or HCP they trust [37, 48]. In addition, most women value conversations about the vaccination issue with others, such as friends, family and peers, which was also the case in our study [25, 26, 34, 44, 51, 52]. Research among social networks indicates that vaccine-hesitant parents include more persons in their social networks related to their decision-making than vaccine-acceptant parents [42].

Before they would make a decision, participants wanted to deliberate over the vaccination issue, guided by personal values they considered to be most important. This study supports evidence from previous findings, showing that vaccine-hesitant parents follow a more thorough deliberation process by carefully weighing the pros, cons, alternatives and consequences of accepting or refusing vaccination, compared to acceptors [36, 49]. There is a relatively small body of literature that emphasizes the deliberation of values in regard to vaccination decision-making [38, 40, 53, 56].

Similar to the orthodox Protestant participants, parents in other studies valued the importance of ‘being a good parent’ by taking responsibility for their child’s health in the context of childhood vaccinations as well [17, 39, 46, 54]. Corresponding to our results, these findings state that taking parental responsibility can result in accepting vaccination to protect a child from disease, or decline vaccination to protect a child from harm caused by the vaccine. Considering the observed critical and proactive attitude of the orthodox Protestant women in this vaccination decision-making process, it is noteworthy that the present generation of orthodox Protestant women follows the rising trend of self-determined parents who want to take responsibility for their child’s health and take a proactive role in their decision-making processes which is seen in many high-income countries [23, 41, 44, 45]. The gendered aspect found in others studies, that women are often the primary decision-makers, was not recognized by our respondents, as they stated that the final vaccine decision-making is done by husband and wife together [44, 55].

As religion plays a central role in the lives of orthodox Protestant women, religious values influenced their vaccination decision and health attitudes in general, which has previously been found in this religious group, as well as in other groups with a religious or spiritual lifestyle [18, 50, 57].

According to some participants in our study, a healthy lifestyle and nutrition could prevent one’s child from disease and, thus, serve as alternatives to vaccinations. Similar health values have been mentioned in relation to vaccination decisions by parents with an anthroposophical lifestyle [17, 39, 43]. The correspondence of our and other findings on the influence of personal values on decision-making and acceptance on vaccination, can indicate that overall, similar to our participants, parents feel a need for value deliberation to make a well-considered decision regarding vaccination.

Earlier research indicates that the involvement of HCPs and religious leaders in the vaccine decision-making process of orthodox Protestant parents, besides providing and explaining medical information (HCP) or biblical principles (religious leaders), is limited [58, 59]. A study among Dutch HCPs indicated that whether or not a HCP discussed vaccine decision-making with orthodox Protestant parents depended on the willingness of these parents to engage in such a discussion and on the personal characteristics and communication skills of the HCP [59]. As HCPs’ communication skills and content knowledge are of great importance to address vaccine hesitancy, this framework can provide HCPs with adequate insight to guide parents who ask advice from this HCP [60]. Further, health policies and finances should be organized as such, that HCPs supporting these parents, have sufficient time, recourses and opportunities to strengthen their vaccination consultations [60, 61].

### Strengths and limitations

To establish a vaccination decision-making framework we used an explorative multimethod approach, combining in-depth interviews and OFGs for data collection with a literature search and research group meetings, to establish possible gaps and refine our preliminary framework. The data-triangulation of interviews and OFGs in combination with the literature search and research group meetings made it possible to first explore the individual decision-making process and, subsequently, gain deeper insight into the presented stages and corresponding needs of decision-making that were shared in a group setting. The group discussions emphasized which needs and opinions were widely supported and which were more individually-based. Additionally, it is expected that the anonymous participation and the possibility for women to respond in their own pace, contributed to the reliability and completeness of the OFG findings. Data validity was further increased by using environmental triangulation, as the interviews were conducted at the participants’ homes and the focus groups in an anonymous online environment.

With regard to limitations, our purposeful sampling resulted in a small, yet, varied study sample of church denomination, age, pregnancy status, vaccination status, and number of children in participants. Data saturation was reached. Due to the small study sample, however, results have to be interpreted with caution. Still, the results give a valuable glimpse into understanding the decision-making process and its related needs in this vaccine-hesitant group. Participants’ education level was not included in our data gathering. It is possible that more higher than lower educated women participated in our study, more highly educated woman in various studies show an intense need for information seeking and reasoned decision-making, similar to the women in our study [41, 45, 62]. On the other hand, other sources indicate that this thorough decision-making process may also characterize (orthodox) Protestants in general [18, 63]. Nevertheless, we recommend future vaccination studies to investigate the influence of education level on decision-making, as well.

Finally, the timing of the data collection was considered to be both a strength and a limitation of the study. This study was conducted before the implementation of the maternal pertussis vaccination in the Netherlands. Therefore, we were able to explore women’s needs for decision-making before their needs were affected by the national immunisation campaign and standardised procedures and interventions. Conducting this study on a hypothetical situation allowed women to be more honest and open about their decision-making without feeling additional social desirability effects. The drawback of this time point of investigation, however, is that women had to hypothesize how they would conduct their decision-making process in the future, which some women found challenging. Future research is required to confirm our findings in a real decision-making situation.

## Conclusion

Our results indicate that, after the Vaccination offer of the maternal pertussis vaccination, Dutch orthodox Protestant women gather information and discuss the vaccination issue with others to orient themselves on the vaccination. Subsequently, the study results imply that women deliberate over the values they consider to be most important in this vaccination decision–parental responsibility, religious values and/or health- to make a well-considered Final decision.

Our framework provides a glimpse into the decision-making process of vaccine-hesitant religious women and can be used to assist HCPs. Alongside providing and explaining information, HCPs could support women, who experience difficulties in vaccine decision-making, by providing additional consultations and adjust their communication to the stages and expressed needs in our framework. Therefore, policymakers and public health institutes should provide HCPs with means and opportunities to meet women’s decision-making needs. Future research that builds upon the results of this study’s findings, accompanied by experiences in clinical practice, can be used to determine in which manner HCPs and other professionals can facilitate women in their different vaccine decision-making stages. As our decision-making framework describes a decision-making process that could be similar to the process of other vaccine-hesitant subgroups, future studies should investigate whether the stages of decision-making in our framework can be found in other vaccine-hesitant subgroups as well.

## Supporting information

S1 Fig Topic guide interviews in Dutch. (PDF)

S1 Table Topic guide online focus groups in Dutch. (PDF)
