# Supplementary material for: Decision-making on maternal pertussis vaccination among women in a vaccine-hesitant religious group: Stages and needs
Source: PLoS One. 2020 Nov 12;15(11):e0242261. doi: 10.1371/journal.pone.0242261 (PMC7660565; doi:10.1371/journal.pone.0242261)
Supplement: S1 Fig — (PDF) [file pone.0242261.s001.pdf]

Topic guide interviews

Opening interview

Algemene vraag over ervaring / kennis over kinkhoest.

Weleens kinkhoest meegemaakt in de omgeving?

Introductie van maternale kinkhoestvaccinatie (MKV)

Heeft u al weleens gehoord over de vaccinatie tegen kinkhoest die vrouwen tijdens hun zwangerschap kunnen krijgen?

ja

Wat weet u over deze vaccinatie?

Zelf aangeboden gekregen?

nee

Korte uitleg over MKV

Eind 2015 heeft de gezondheidsraad het advies uitbracht om elke zwangere vrouw in Nederland de maternale kinkhoestvaccinatie aan te bieden. De vaccinatie wordt gegeven tussen de 28 en 32 weken zwangerschap. Door de vaccinatie gaat het lichaam van de zwangere vrouw antistoffen aanmaken tegen kinkhoest. Deze antistoffen komen via de navelstreng bij de baby terecht. Bij de geboorte heeft de baby voldoende antistoffen om beschermd te zijn tegen kinkhoest tijdens de eerste maanden van zijn/haar leven.

Wat is uw eerste reactie nu ik dit vertel?

Besluitvorming

Proces

Stel, u krijgt de vaccinatie aangeboden van de verloskundige.

Hoe zou u een besluit nemen?

Bespreekt u de vaccinatie(keuze) met anderen?

Wie / wanneer?

Welke rol heeft deze ander?

Wat heeft u nodig om besluit te nemen?

Wat zou u helpen om een besluit te kunnen nemen?

Waar zou u behoefte aan hebben?

Welke anderen kunnen u daarin ondersteunen?

indien informatiebehoefte; doorvragen hoe/wat/wanneer?

Hoe komt u tot een uiteindelijk besluit?

Wat heeft u nodig om tot een uiteindelijke ja of nee te komen?

Moeilijke beslissing?
