# Supplementary material for: Decision-making on maternal pertussis vaccination among women in a vaccine-hesitant religious group: Stages and needs
Source: PLoS One. 2020 Nov 12;15(11):e0242261. doi: 10.1371/journal.pone.0242261 (PMC7660565; doi:10.1371/journal.pone.0242261)
Supplement: S1 Table — (PDF) [file pone.0242261.s002.pdf]

Topic Guide Online Focus Groups

Dag 1: Informatie

Vraag van de dag

U ontving van ons informatie over de kinkhoestvaccinatie voor zwangere vrouwen.

- Wat was uw eerste reactie op de informatie? Wat vond u goed aan de informatie? En wat vond u minder goed aan de informatie?

*Aanvullende vragen**

- Zou u na het lezen van de informatie op zoek gaan naar aanvullende informatie? Zo ja, waar en welke informatie?
- Zou u -na het lezen van de meegestuurde informatie- een besluit kunnen nemen om u wel of niet te laten vaccineren? Kunt u toelichting waarom u al wel of nog geen besluit kunt nemen?
- Zou u informatie wensen vanuit de reformatorische achterban?
- Zou u informatie willen ontvangen die gaan over de keuze om niet te vaccineren?
- Stel, er bestaat een website met een keuzehulp om u te helpen met het nemen van een besluit om wel of niet te vaccineren. Zou u hiervan gebruik willen maken?

Dag 2: Zorgverleners en anderen

Vraag van de dag

Stel, u bent zwanger en de verloskundige benoemt dat u een kinkhoestvaccinatie kunt krijgen.

- Hoe zou de verloskundige u kunnen ondersteunen in het nemen van een besluit om uzelf wel of niet te laten vaccineren?

*Aanvullende vragen**

- Zou u ook een persoonlijk advies willen krijgen van uw verloskundige?
- Welke ondersteuning zou u eventueel nog van een andere zorgverlener willen krijgen?
- Zou u met anderen willen praten over de kinkhoestvaccinatie (anders dan de verloskundige)? En aan welke personen denkt u dan? Begint u zelf over dit onderwerp met hen?
- […] geeft aan dat ze de vaccinatie ook met haar man zou bespreken. Hoe is dat voor de anderen?
- Zou u de vaccinatiekeuze met personen willen bespreken met gelijke geloofsachtergrond?

Dag 3: Geloofsovertuiging

Christenen betrekken God of hun geloofsovertuiging vaak bij het nemen van een besluit.

- Speelt bij u uw geloofsovertuiging een rol bij het nemen van een besluit over de kinkhoestvaccinatie tijdens de zwangerschap? Kunt u uw antwoord toelichten?

Aanvullende vragen

- Maakt u er een moment voor vrij om uw geloofsovertuiging te betrekken bij het maken van uw besluit?
- Speelt bij u uw gevoel of uw levenshouding een rol bij het nemen van een besluit?

Dag 4: Overweging besluit

Uit eerdere berichten maak ik op dat de meesten van u -om een besluit te kunnen nemen- verschillende onderdelen afwegen. Met onderdelen bedoel ik bijvoorbeeld het lezen en verzamelen van informatie, in gesprek gaan met uw echtgenoot en (eventueel) in gesprek gaan vrienden, familie en verloskundige. Sommigen van u geven aan de overweging om wel of niet te vaccineren mee te nemen in gebed of te betrekken bij het lezen uit de Bijbel.

- Hoe weegt u de verschillende onderdelen tegen elkaar af bij deze vaccinatie? en
- Kunt u toelichten waar het uit voortkomt dat u een goede afweging wil maken?

*Aanvullende vragen**

- Heeft bij u de wens om een overwogen besluit te maken te maken met een gevoel van verantwoordelijkheid?
- Zou een gesprek met anderen kunnen bijdragen aan het nemen van een besluit?

Dag 5: Evaluatie focusgroep

Vraag van de dag

Op dit forum heeft u de afgelopen dagen met andere vrouwen gepraat over de kinkhoestvaccinatie voor zwangere vrouwen.

- Wat vond u van de online gespreksgroep?
- Zou u deze manier van online praten met anderen u helpen als u een besluit over deze vaccinatie zou moeten nemen? Kunt u dit toelichten?

*Aanvullende vragen**

- Op vraag 1: Wat vond u prettig of minder prettig aan de online gespreksgroep?
- Van alles waar over gesproken is op het forum; wat was voor u het belangrijkste onderwerp?
- Heeft u het idee dat we nog iets vergeten zijn om te bespreken?

Stel dat er vanuit de verloskundige praktijk een groepsbijeenkomst georganiseerd wordt over kinkhoestvaccinatie tijdens de zwangerschap. Bij deze bijeenkomst wordt er informatie gegeven en u krijgt de gelegenheid om vragen te stellen en om met andere vrouwen in gesprek te gaan.

- Zou u aan zo een bijeenkomst deel willen nemen? Zou u uw antwoord kunnen toelichten?
- Zou het voor uzelf een verschil maken of zo een bijeenkomst georganiseerd wordt door een christelijke organisatie (bv. NPV)

* Aanvullende vragen. Gedurende de OFG-dag kon uit de aanvullende vragen een doorvraag, passend bij de online conversatie, gekozen worden. Per dag werden er gemiddeld 2 aanvullende vragen gesteld.
